# From Array-CGH to Whole-Genome Sequencing: A 29-Year Diagnostic Journey Culminating in the Identification of a De Novo ABCC9 Variant Consistent with Cantú Syndrome

**DOI:** 10.3390/diagnostics16142204

**Published:** 2026-07-15

**Authors:** Chung-Lin Lee, Ya-Hui Chang, Chih-Kuang Chuang, Huei-Ching Chiu, Yuan-Rong Tu, Yun-Ting Lo, Jun-Yi Wu, Hsiang-Yu Lin, Shuan-Pei Lin

**Affiliations:** 1 Department of Pediatrics, MacKay Memorial Hospital, Taipei 104217, Taiwan; clampcage@gmail.com (C.-L.L.); wish1001026@gmail.com (Y.-H.C.); g880a01@mmh.org.tw (H.-C.C.); 2 Institute of Clinical Medicine, National Yang-Ming Chiao-Tung University, Taipei 112304, Taiwan; 3 International Rare Disease Center, MacKay Memorial Hospital, Taipei 104217, Taiwan; andy11tw.e347@mmh.org.tw (Y.-T.L.); wl01723138@gmail.com (J.-Y.W.); 4 Department of Medicine, MacKay Medical University, New Taipei City 252005, Taiwan; 5 Department of Nursing, MacKay Junior College of Medicine, Nursing and Management, New Taipei City 252005, Taiwan; 6 Division of Genetics and Metabolism, Department of Medical Research, MacKay Memorial Hospital, Taipei 104217, Taiwan; mmhcck@gmail.com (C.-K.C.); likemaruko@hotmail.com (Y.-R.T.); 7 College of Medicine, Fu-Jen Catholic University, New Taipei City 242062, Taiwan; 8 Department of Medical Research, China Medical University Hospital, Taichung 404327, Taiwan; 9 Department of Infant and Child Care, National Taipei University of Nursing and Health Sciences, Taipei 112303, Taiwan

**Keywords:** Cantú syndrome, *ABCC9*, K_ATP_ channel, hypertrichosis, whole-genome sequencing, diagnostic odyssey, NGS reanalysis, rare disease

## Abstract

**Background and Clinical Significance**: Cantú syndrome (OMIM #239850) is a rare autosomal dominant disorder caused by gain-of-function variants in *ABCC9* or *KCNJ8*, which encode subunits of the ATP-sensitive potassium (K_ATP_) channel. Its characteristic features—generalized hypertrichosis, coarse facial appearance, skeletal abnormalities, and cardiovascular involvement—may be overlooked when other major comorbidities dominate the clinical picture. **Case Presentation**: A 29-year-old Taiwanese woman, born prematurely and complicated by neonatal hydrocephalus with subdural hemorrhage requiring ventriculoperitoneal shunt placement, had been followed since infancy under a working diagnosis of cerebral palsy with left hemiparesis and borderline-to-mild intellectual disability. Over the ensuing years, additional features gradually emerged, including generalized hypertrichosis with thick scalp and body hair, coarse facial features, bilateral hallux valgus, mild thoracic scoliosis, polycystic ovaries, mild aortic regurgitation, recurrent hemoptysis associated with abnormal pulmonary vasculature, and iron-deficiency anemia. Earlier genetic investigations—including chromosome analysis (46,XX), array comparative genomic hybridization (array-CGH; 2013), and a trio-based next-generation sequencing study performed under a national rare disease research initiative (2019)—were unrevealing. Whole-genome sequencing performed in December 2025 identified a heterozygous *ABCC9* variant (NM_020297.4:c.4174A>G, p.(Ile1392Val)), initially classified as a variant of uncertain significance. Parental Sanger sequencing confirmed the variant to be de novo, and reclassification according to ACMG/AMP criteria supported a likely pathogenic interpretation. Re-evaluation of the patient’s phenotype demonstrated findings consistent with Cantú syndrome. **Conclusions**: This case illustrates how Cantú syndrome may remain unrecognized for years when a prominent neurological comorbidity—perinatally acquired hydrocephalus and presumed cerebral palsy—dominates the clinical narrative. We report a previously undescribed de novo *ABCC9* missense variant (c.4174A>G, p.(Ile1392Val)), thereby expanding the mutational spectrum associated with Cantú syndrome. This case also highlights the practical value of resequencing and periodic reanalysis using updated next-generation sequencing platforms in patients with long-standing undiagnosed disease, even after prior negative genetic testing.

## 1. Introduction

Cantú syndrome (CS; OMIM #239850) was first described in 1982, when Cantú and colleagues reported a Mexican family with congenital generalized hypertrichosis, coarse facial features, cardiomegaly, and distinctive osteochondrodysplasia [1]. Over the subsequent four decades, approximately 150 cases have been reported worldwide [2,3], and the recognized phenotype has expanded considerably beyond the original triad. The largest molecularly confirmed cohort to date, comprising 74 patients, found hypertrichosis and coarse facial features to be nearly universal. Cardiomegaly was present in 64% of patients, patent ductus arteriosus in 58%, and pericardial effusion in 25%. Additional manifestations included macrosomia, macrocephaly, peripheral lymphedema, pulmonary hypertension, vascular tortuosity, and mild intellectual impairment in a subset of patients [3,4]. More recently, the cardiovascular phenotype has been reinterpreted as high-output cardiac hypertrophy driven by genetically determined low systemic vascular resistance, thereby unifying previously disparate cardiac, vascular, and hemodynamic findings [4].

The molecular basis of the syndrome remained unknown for nearly three decades. In 2012, two independent groups identified heterozygous gain-of-function (GoF) missense variants in *ABCC9* as the major cause of CS [5,6]. *ABCC9* encodes the regulatory sulfonylurea receptor 2 (SUR2) subunit of ATP-sensitive potassium (K_ATP_) channels. A smaller proportion of patients harbor analogous GoF variants in *KCNJ8*, which encodes the pore-forming Kir6.1 subunit [7]. The shared functional consequence is reduced ATP-mediated inhibition of channel gating, often accompanied by enhanced Mg-nucleotide-dependent activation, resulting in abnormally increased K_ATP_ channel opening in tissues expressing SUR2-containing channels, including vascular smooth muscle, cardiac and skeletal muscle, and hair follicles [8,9]. Reported variants are not uniformly distributed throughout the protein but instead cluster within or near the transmembrane domains (TMDs) and the two intracellular nucleotide-binding domains (NBDs). Electrophysiological studies have demonstrated that variants in different regions produce GoF effects through distinct mechanisms [8,10]. More recently, biallelic loss-of-function variants in the same gene were shown to cause a partially contrasting phenotype termed *ABCC9*-related intellectual disability and myopathy syndrome (AIMS; OMIM #619719) [11,12].

Despite advances in molecular understanding, clinical recognition of CS remains challenging. No formal diagnostic criteria have been established [3]. The cardinal manifestations overlap substantially with those of Zimmermann–Laband syndrome [13] and Coffin–Siris syndrome, while additional differential diagnoses include mucopolysaccharidoses and acromegaloid facial appearance syndrome [3]. Even hypertrichosis, often the most recognizable feature, is nonspecific and may be attributed to constitutional or ethnic variation, endocrine disorders, or medication exposure such as minoxidil [3]. Diagnostic difficulty is further compounded when unrelated major comorbidities dominate the clinical presentation from infancy. In such cases, the manifestations of CS may gradually accumulate over time and become incorporated into the primary diagnosis rather than recognized as features of a separate syndrome.

For such patients, periodic genomic reanalysis or resequencing using updated platforms has emerged as a valuable diagnostic strategy. Reanalysis of clinical exome sequencing data in a large diagnostic cohort yielded additional molecular diagnoses in approximately 10% of previously undiagnosed patients [14]. More recent large-scale automated reanalysis programs achieved new diagnoses in 5–6% of previously unsolved cases, largely owing to newly established gene–disease associations and improved bioinformatic approaches [15]. In pediatric rare disease cohorts, whole-genome sequencing provides an additional diagnostic yield of approximately 7% beyond that of exome sequencing alone [16].

The present patient illustrates both aspects of this diagnostic challenge. She is a 29-year-old Taiwanese woman born prematurely and complicated by neonatal hydrocephalus with subdural hemorrhage requiring ventriculoperitoneal shunt placement. Since infancy, she had been followed under a working diagnosis of cerebral palsy with left hemiparesis and borderline-to-mild intellectual disability. Over the following decades, multiple features highly suggestive of CS gradually became apparent, including pronounced generalized hypertrichosis, coarse facial appearance, bilateral hallux valgus, thoracic scoliosis, polycystic ovaries, mild aortic regurgitation, and recurrent hemoptysis associated with abnormal pulmonary vasculature. However, these findings were never integrated into a unified syndromic diagnosis. Earlier genetic investigations, including chromosome analysis, array comparative genomic hybridization, and trio-based next-generation sequencing performed in 2019 under a national rare disease research initiative, were unrevealing. Whole-genome sequencing performed in late 2025 identified a heterozygous *ABCC9* variant (NM_020297.4:c.4174A>G, p.(Ile1392Val)). Parental Sanger sequencing confirmed the variant to be de novo, and ACMG/AMP classification supported a likely pathogenic interpretation. This variant has not previously been reported in ClinVar, HGMD, or the published literature. The affected residue is located within the second nucleotide-binding domain (NBD2) of SUR2, a region in which relatively few disease-associated variants have been reported.

## 2. Case Presentation

### 2.1. Patient and Clinical History

The proband is a 29-year-old Taiwanese woman and the only child of healthy, nonconsanguineous parents. The family history was unremarkable for hypertrichosis, congenital heart disease, syndromic intellectual disability, or other conditions overlapping with Cantú syndrome.

She was born preterm at approximately 32 weeks of gestation after a pregnancy that, on review of the available obstetric records, was not documented to have been complicated by polyhydramnios, with a birth weight of approximately 2800 g, which was large for gestational age. Her neonatal course was complicated by hydrocephalus with subdural hemorrhage. A ventriculoperitoneal (VP) shunt was placed during early infancy. In October 2006, partial resection of the distal shunt tubing was performed because of shunt-related complications, and an Ommaya reservoir was subsequently placed in the right parieto-occipital region, where it has remained in situ.

From childhood, she was followed under a working diagnosis of cerebral palsy with left-sided spastic hemiparesis, left claw-hand deformity, and joint contractures involving all major joints on the left side, together with borderline-to-mild intellectual disability. She completed a special education program through high school in 2013 and, since 2022, has participated in a sheltered workshop program. Additional medical history included a mid-shaft fracture of the left humerus treated with internal fixation, which remained visible on chest radiography, and a methicillin-resistant *Staphylococcus aureus* (MRSA)-positive open wound infection over the back in 2014.

**Figure 1:**
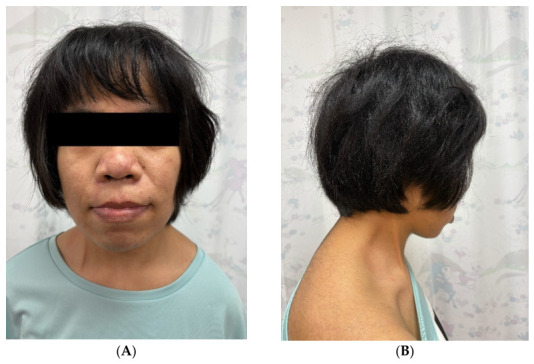
Craniofacial features of the proband. (**A**) Frontal view showing coarse facial appearance with a broad nasal bridge, full lips, fine facial hypertrichosis over the cheeks with a faint mustache, and mild rosacea-like erythema with periorificial folliculitis over the nasal and perinasal regions. (**B**) Right lateral view demonstrating abundant scalp hair extending onto the neck and shoulders, a low posterior hairline, and irregularity of the posterior occipital contour related to the previously placed ventriculoperitoneal shunt. A black bar has been placed across the eyes to preserve patient anonymity.

**Figure 2:**
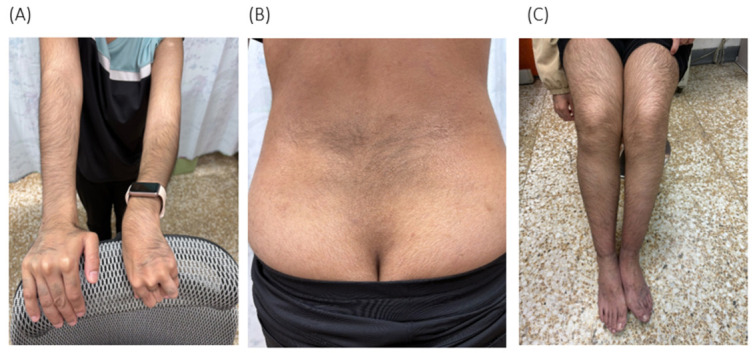
Composite documentation of generalized hypertrichosis. (**A**) Bilateral forearms and dorsum of the hands showing dense, dark terminal hair extending distally to the proximal phalanges. (**B**) Lower back and sacral region demonstrating a midline whorl-like pattern of hypertrichosis. (**C**) Lower extremities showing thick, abundant dark terminal hair covering both thighs and lower legs, with extension onto the ankles and dorsum of the feet.

Over time, multiple features suggestive of an underlying syndromic diagnosis gradually accumulated but were not recognized collectively at the time. These included progressive generalized hypertrichosis with thick and abundant scalp and body hair (Figure 1 and Figure 2), coarse facial features, bilateral hallux valgus (more severe on the left side), mild thoracic scoliosis with a positive Adam’s forward bend test, and polycystic-appearing ovaries on transabdominal ultrasonography (right ovary, 1.75 × 2.5 cm; left ovary, 1.86 × 2.59 cm, with multiple follicle-like structures). She also had oligomenorrhea, with menstrual cycles occurring approximately every 5–6 weeks, and previously heavy menstrual flow that had recently decreased to light bleeding lasting 1–2 days. Additional findings included iron-deficiency anemia requiring intermittent supplementation, mild aortic regurgitation on serial echocardiography, intermittent peripheral edema with bruise-like discoloration over the left ankle, and a notable episode of recurrent moderate-to-massive hemoptysis in 2012. Imaging at that time suggested abnormal pulmonary vasculature with multiple sites of extravasation.

### 2.2. Phenotypic Features at the Most Recent Assessment

At the most recent clinic visit in May 2026, the patient’s height was 160.1 cm and weight was 57.7 kg (body mass index, 22.5 kg/m^2^). She appeared pink and well oxygenated while breathing room air. Examination findings are summarized below by organ system.

#### 2.2.1. Skin, Hair, and Adnexa

**Figure 3:**
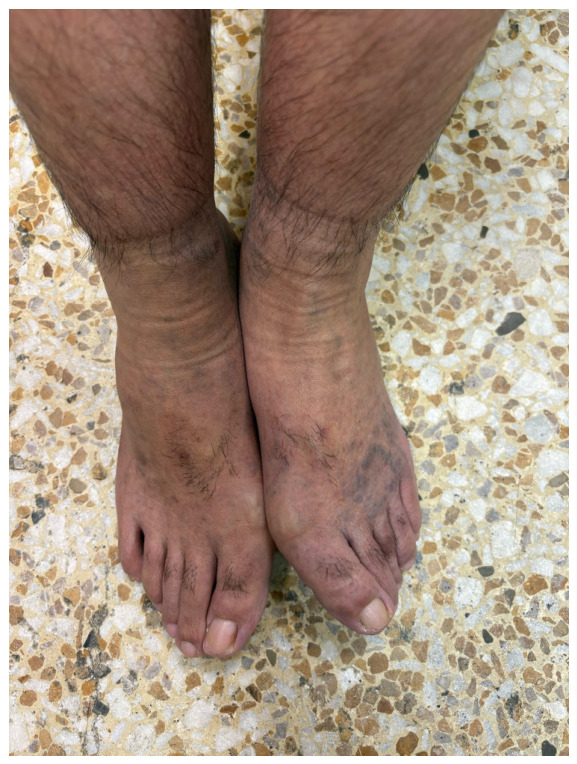
Feet of the proband demonstrating multiple manifestations of Cantú syndrome. These findings include: (i) hypertrichosis extending onto the dorsum of both feet and toes; (ii) bilateral hallux valgus, more severe on the left side; and (iii) diffuse hyperpigmentation and bruise-like discoloration over the left ankle and dorsal foot, with freckle-like pigmentation, consistent with the lymphatic and vascular involvement described in Cantú syndrome.

Generalized hypertrichosis was the most prominent physical finding. Thick, abundant scalp hair extended onto the forehead, resulting in a low frontal hairline. Fine-to-coarse facial hair was present over the cheeks, accompanied by a mild mustache. Dense terminal hair covered the arms and the dorsum of the hands (Figure 2A) and formed a midline whorl-like distribution over the lower back and sacral region (Figure 2B). The thighs and lower legs were also covered with dense, dark terminal hair extending onto the dorsum of the feet and toes (Figure 2C and Figure 3).

Several small acquired nevi were observed over the abdominal wall and extremities, and a 0.45 cm pigmented raised nevus was noted on the right earlobe. The face demonstrated acneiform eruptions with mild rosacea and periorificial folliculitis. A small, soft, mobile subcutaneous nodule was palpable over the right ankle. The left ankle showed freckle-like pigmentation with diffuse bruise-like discoloration (Figure 3), findings consistent with the lymphatic and vascular involvement described in Cantú syndrome.

#### 2.2.2. Craniofacial

Coarse facial features were evident, including a broad nasal bridge, full vermilion, mild macroglossia, and a high-arched palate. Mild pharyngeal injection without exudate was noted. Bilateral cerumen impaction was present. The occipital contour was irregular, with a palpable Ommaya reservoir in the right parieto-occipital region and a VP shunt tube traceable subcutaneously along the right side of the neck. A small, soft mass was palpable over the occipital region. No acute facial nerve palsy was observed, although mild dysarthria related to prior central nervous system injury persisted.

#### 2.2.3. Cardiopulmonary

A grade 1/6 soft systolic murmur was audible at the left lower sternal border. Cardiac rhythm was regular at 82 beats/min. Blood pressure had historically been labile, with intermittent borderline elevation documented during outpatient follow-up, but was well controlled at the most recent visit without antihypertensive therapy. The most recent transthoracic echocardiogram (August 2024) demonstrated trivial aortic regurgitation with preserved left ventricular systolic function; left ventricular outflow tract velocity was normal (153 cm/s). On quantitative assessment, the ejection fraction was 81%, with left ventricular end-diastolic and end-systolic internal dimensions of 51.6 mm and 25.8 mm. Interventricular septal and posterior wall thicknesses were normal at 7.9 mm and 8.9 mm, with a left ventricular mass of 153 g (mass index, 98 g/m^2^ by the cube method); relative wall thickness was normal, and there was no left ventricular hypertrophy or chamber dilatation. The aortic root was of normal caliber (22.1 mm), and a left aortic arch without coarctation was confirmed. Trivial tricuspid regurgitation was present (peak gradient 26 mmHg) without mitral regurgitation, and no pericardial effusion was reported.

Lung fields were clear on auscultation, and no dyspnea was present. Chest radiography obtained in October 2024 demonstrated clear costophrenic angles, an unremarkable mediastinal and cardiac silhouette, mild thoracic scoliosis, and postoperative internal fixation changes involving the left humerus.

#### 2.2.4. Musculoskeletal and Neurological

Hypoplasia and spasticity of the left-sided limbs were present, accompanied by joint contractures involving all major joints on the left side and a left claw-hand deformity. Muscle strength was Medical Research Council grade 4–5/5 in the left upper and lower extremities and 5/5 on the right side. Bilateral hallux valgus was observed and was more pronounced on the left (Figure 3). Adam’s forward bend test was positive, consistent with mild thoracic scoliosis confirmed radiographically. Cognitive function remained within the borderline-to-mild intellectual disability range, accompanied by slurred speech.

#### 2.2.5. Abdominal and Genitourinary

The abdomen was soft and nondistended, without organomegaly or umbilical hernia. Pubic hair distribution was normal. Renal ultrasonography performed in July 2024 demonstrated bilaterally increased renal echogenicity with reduced corticomedullary differentiation, as well as two small subcapsular cysts in the right kidney, the largest measuring 3.4 × 5.5 mm. Concurrent biochemical and urinalysis findings were within normal limits.

Abdominal ultrasonography performed in January 2026 confirmed bilateral ovarian cystic lesions consistent with polycystic-appearing ovaries (right ovary, 1.75 × 2.5 cm; left ovary, 1.86 × 2.59 cm), most likely representing follicles.

#### 2.2.6. Selected Laboratory Findings

Recent complete blood count results showed a hemoglobin level of 13.7 g/dL with normocytic indices (mean corpuscular volume [MCV], 88.4 fL; mean corpuscular hemoglobin concentration [MCHC], 32.8 g/dL). Ferritin was 18.12 ng/mL. A previous episode of microcytic iron-deficiency anemia in January 2025 (hemoglobin, 10.3 g/dL; MCV, 78.7 fL; serum iron, 13 µg/dL; total iron-binding capacity [TIBC], 391 µg/dL; ferritin, 3.09 ng/mL) improved after oral iron supplementation.

A reproductive hormone panel obtained in January 2025 showed follicle-stimulating hormone (FSH) 6.48 mIU/mL, estradiol 28.27 pg/mL, progesterone 0.1 ng/mL, testosterone 0.29 ng/mL, androstenedione 0.80 ng/mL, and dehydroepiandrosterone sulfate (DHEA-S) 92.36 µg/dL, all within reference ranges for reproductive-age women. Anti-HBs antibody titer was protective at 64.09 mIU/mL.

### 2.3. Past Genetic Workup

Three rounds of genetic testing were performed before the current diagnosis was established. Conventional karyotyping performed in December 2006 demonstrated a normal female karyotype (46,XX) without detectable chromosomal abnormalities. Array comparative genomic hybridization performed in November 2013 revealed no clinically significant copy number variants. Subsequently, a trio-based next-generation sequencing study performed in June 2019 under a national rare disease research initiative focused on intellectual disability and multiple congenital anomalies, identified no pathogenic or likely pathogenic variant. Because this was an internal research initiative rather than a clinical diagnostic test, the program retained its detailed gene content, variant-filtering criteria, and bioinformatic pipeline and did not release them to the referring team; the exact gene set and analytic workflow used in 2019 therefore cannot be specified here.

Neither *ABCC9* nor Cantú syndrome had been specifically considered during these earlier evaluations. During this period, the clinical picture remained dominated by cerebral palsy and shunt-related neurological sequelae, whereas the syndromic features that later proved diagnostically informative accumulated only gradually over time.

### 2.4. Whole-Genome Sequencing and Confirmation

In December 2025, following continued accumulation of clinical findings and renewed multidisciplinary evaluation, the patient was enrolled in a clinical whole-genome sequencing program. WGS was performed using peripheral blood genomic DNA obtained from the proband at Kim Forest Laboratory (New Taipei City, Taiwan). Sequencing was conducted using the Illumina DNA PCR-Free Prep with Tagmentation library preparation kit on an Illumina NovaSeq X Plus platform (Illumina, Inc., San Diego, CA, USA) with paired-end 2 × 151 bp reads. Mean sequencing depth was 35×, with ≥10× coverage achieved across 93.55% of the genome. Reads were aligned to the GRCh38 reference genome, and variants were called using DRAGEN version 4.2.6. Variant annotation incorporated OMIM (2024-12), ClinVar (2024-12), gnomAD-exomes v2.1.1, gnomAD-mitochondrial v2.1.1, and dbSNP build 1405. Variant interpretation followed the American College of Medical Genetics and Genomics/Association for Molecular Pathology (ACMG/AMP) guidelines. Parental Sanger sequencing for de novo confirmation was subsequently performed as a separate analysis.

**Figure 4:**
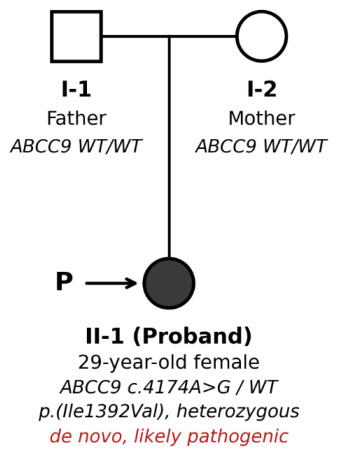
Pedigree of the proband and her parents. Both parents (I-1 and I-2) carry wild-type *ABCC9* alleles. The proband (II-1; arrow) is heterozygous for the *ABCC9* c.4174A>G [p.(Ile1392Val)] variant, which was confirmed to have arisen de novo.

**Figure 5:**
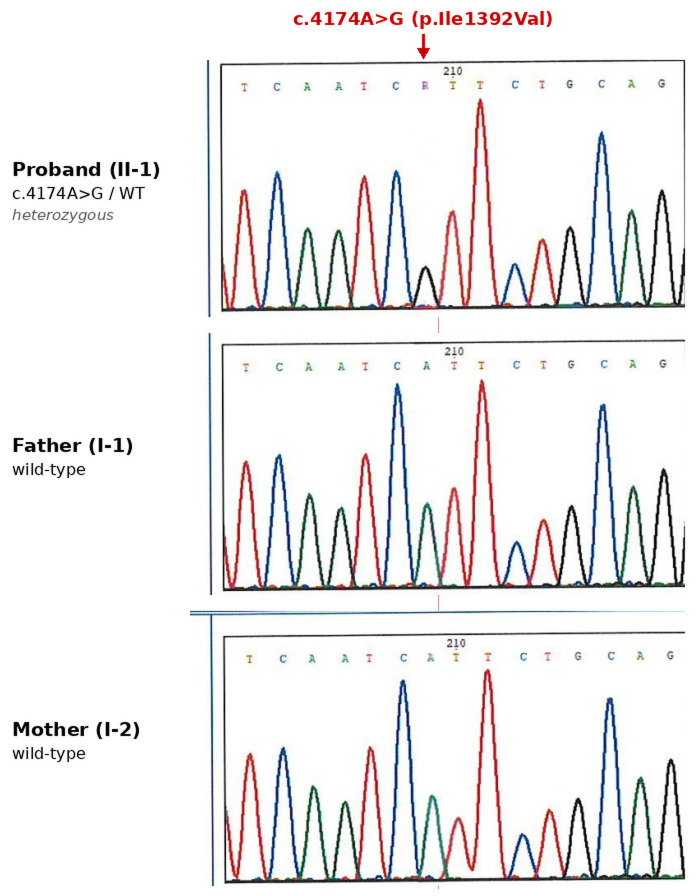
Sanger sequencing electropherograms confirming the de novo status of the *ABCC9* c.4174A>G variant. Both parents (**middle panel**, **lower panel**) demonstrate homozygous wild-type peaks (A, green) at the variant position. The proband (**top panel**) shows overlapping A and G peaks at the same position, consistent with heterozygosity.

A heterozygous *ABCC9* variant (NM_020297.4:c.4174A>G; NC_000012.12:g.21812086T>C, GRCh38) was identified in the proband and was absent in both parents (Figure 4 and Figure 5). The variant is predicted to result in a missense substitution, p.(Ile1392Val), affecting the sulfonylurea receptor 2 (SUR2) protein. Because residue 1392 lies within the shared SUR2 core, N-terminal to the alternatively spliced C-terminal exon that distinguishes the two major isoforms, the substitution is predicted to affect both the SUR2A and SUR2B isoforms. Targeted Sanger sequencing performed on parental and proband DNA in March 2026 confirmed the variant to be de novo, with maternity and paternity formally established.

The variant has not been reported in ClinVar, the Human Gene Mutation Database, or the Leiden Open Variation Database for *ABCC9*, and was absent from gnomAD-exomes v2.1.1, which was used in the diagnostic pipeline. Independent interrogation of gnomAD v4.1.1 (~807,000 individuals across exome and genome datasets) confirmed continued absence of the variant despite adequate sequence coverage (29.9× in exomes and 30.9× in genomes). The variant was also absent from Taiwan BioBank and dbSNP.

In silico prediction results were mixed but overall favored a deleterious effect. REVEL yielded a score of 0.684, falling within the moderate threshold range (0.644–0.772) proposed by the ClinGen Sequence Variant Interpretation Working Group for PP3 application. Additional prediction scores included a CADD PHRED score of 25.4 (top 0.3% of all possible variants), PrimateAI-3D score of 0.84 (above the *ABCC9*-specific threshold of 0.59), EVE score of 0.6284, and AlphaMissense score of 0.4798, classified as uncertain. SIFT classified the variant as damaging (maximum SIFT score, 0.006), whereas PROVEAN predicted a neutral effect. A BLOSUM100 substitution score of 4 reflected the chemically conservative nature of the isoleucine-to-valine substitution. The affected residue demonstrated strong evolutionary conservation (phyloP100 = 7.597). SpliceAI predicted no significant effect on splicing (maximum Δ score, 0.02).

**Figure 6:**
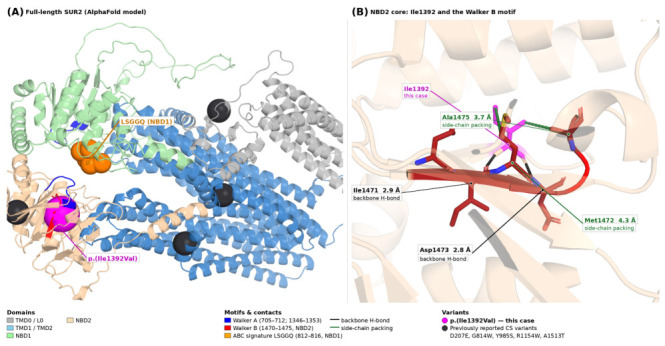
Structural localization of p.(Ile1392Val) in SUR2. Predicted structure of human SUR2 (UniProt O60706; NM_020297.4/NP_064693.2; 1549 amino acids) from the AlphaFold Protein Structure Database. (**A**) Whole-protein cartoon colored by domain: TMD0 and L0, gray; TMD1 and TMD2, light blue; NBD1, light green; NBD2, wheat. Functional motifs are highlighted as follows: the Walker A motifs (residues 705–712 and 1346–1353), blue; the NBD2 Walker B motif (residues 1470–1475), red; and the ABC signature motif (LSGGQ; residues 812–816), orange. The signature motif lies in NBD1, approximately 20 Å from Ile1392. The variant identified in this case, p.(Ile1392Val), is shown as a magenta sphere (arrow); previously reported Cantú syndrome–associated variants are shown as dark gray spheres. (**B**) Close-up of the NBD2 core. The NBD2 cartoon is shown in wheat with the Walker B motif in red. Ile1392 is displayed as magenta sticks and the contacting Walker B residues as dark red sticks; within these side chains, heteroatoms follow standard CPK coloring (nitrogen, blue; oxygen, red; sulfur, yellow). Ile1392 lies on a β-strand that pairs with the Walker B strand through backbone hydrogen bonds (black dashed lines; Ile1392 N–Ile1471 O, 2.9 Å; Ile1392 O–Asp1473 N, 2.8 Å), and its side chain is buried in the hydrophobic core, packing against the Walker B residues Ala1475 and Met1472 (green dashed lines; 3.7 and 4.3 Å, respectively) as well as Leu1470, Gln1455, and Leu1459. The residue makes no direct contact with the catalytic carboxylates of Asp1473 or Glu1474. The model is a computational prediction (per-residue confidence at Ile1392, pLDDT 89.8) and is shown to indicate the structural context of the variant; it does not constitute evidence of a functional effect. Comparison variants are renumbered to NM_020297.4 / NP_064693.2 for internal consistency, with the originally published designations given in parentheses where these differ: D207E, G814W, Y985S (published as Y981S), R1154W (published as R1150W), and A1513T.

The affected residue, Ile1392, lies within the second nucleotide-binding domain (NBD2) of SUR2. In the AlphaFold model of SUR2 (UniProt O60706), Ile1392 sits on a β-strand that pairs, through backbone hydrogen bonds, with the strand carrying the Walker B motif (residues 1470–1475): the amide of Ile1392 hydrogen bonds to the carbonyl of Ile1471 (2.9 Å), and its own carbonyl to the amide of Asp1473 (2.8 Å). Its side chain is buried in the hydrophobic core of NBD2 and packs against the Walker B residues Ala1475 (3.7 Å), Leu1470 (4.2 Å), and Met1472 (4.3 Å), as well as Gln1455 and Leu1459 (Figure 6). It does not contact the catalytic carboxylates of Asp1473 or Glu1474 directly. The ABC signature motif of SUR2 (LSGGQ, residues 812–816) lies in NBD1, approximately 20 Å away, and pairs with the Walker A and Walker B motifs of NBD2 to form the composite nucleotide-binding site. Ile1392 therefore occupies the hydrophobic core immediately beneath the Walker B motif rather than a nucleotide-contacting position itself. We emphasize that Ile1392 has not been shown experimentally to be functionally critical, and no data exist for substitutions at this position. The localization is consistent with, but does not establish, an effect on nucleotide handling at NBD2.

The laboratory initially classified the variant as a variant of uncertain significance because of its absence from population databases at the time of reporting and the limited range of in silico tools included in the diagnostic pipeline. Following confirmation of de novo occurrence by parental Sanger sequencing, independent interrogation of additional meta-predictors recommended by the ClinGen Sequence Variant Interpretation Working Group, and phenotype correlation demonstrating features highly specific for Cantú syndrome, the variant was reclassified according to the ACMG/AMP 2015 guidelines.

The following criteria were applied. PS2 was assigned on the basis of confirmed de novo occurrence with documented maternity and paternity in a patient whose phenotype fits a well-established gene–disease relationship. PM2_supporting reflects absence from gnomAD-exomes v2.1.1, gnomAD v4.1.1 (~807,000 individuals), Taiwan BioBank, and dbSNP, at coverage adequate to detect the variant had it been present. PP3 was applied at moderate strength on the basis of a REVEL score of 0.684, which falls within the moderate range (0.644–0.772) defined by the ClinGen Sequence Variant Interpretation Working Group, together with a CADD PHRED score of 25.4, a PrimateAI-3D score of 0.84 above the *ABCC9*-specific threshold, and strong evolutionary conservation of the residue (phyloP100 = 7.597). PP4 was applied because the phenotype—generalized hypertrichosis with coarse facial features and cardiovascular involvement—is highly specific for Cantú syndrome. In combination, one strong, one moderate, and two supporting criteria meet the ACMG/AMP rule for a likely pathogenic classification.

We acknowledge that this classification is not the only defensible one. The in silico evidence is only partially concordant: PROVEAN predicted a neutral effect, AlphaMissense returned an uncertain score (0.4798), and the BLOSUM100 score of 4 reflects how chemically conservative an isoleucine-to-valine change is—arguments that another laboratory could reasonably use to withhold PP3, or to apply it only at supporting strength. Doing so would leave PS2, PM2_supporting, and PP4, which would yield a variant of uncertain significance rather than a likely pathogenic call. No functional data are available to resolve this, and PS3 cannot be invoked. We have adopted the likely pathogenic classification because the de novo occurrence, the absence from large population datasets, and the phenotypic specificity together provide, in our judgment, sufficient evidence; we recognize that the interpretation rests on this combination rather than on any single decisive line of evidence, and that reclassification in either direction remains possible as evidence accumulates.

### 2.5. Diagnosis

**Figure 7:**
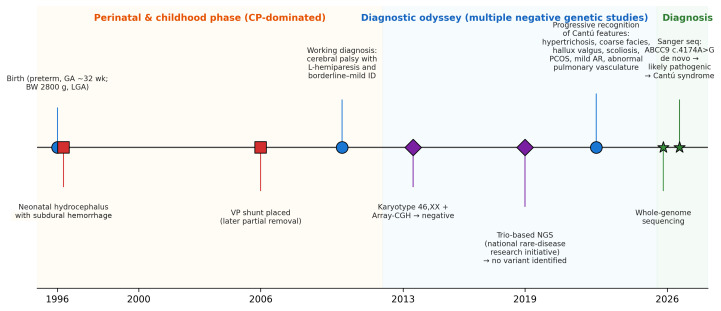
Diagnostic timeline of the proband from preterm birth in 1996 to molecular confirmation of Cantú syndrome in 2026. Events are plotted on a common time axis, with marker shape indicating the type of event: blue circles denote clinical and phenotypic milestones (preterm birth, the working diagnosis of cerebral palsy, and the progressive recognition of Cantú syndrome features); red squares denote neurosurgical events (neonatal hydrocephalus with subdural hemorrhage, and ventriculoperitoneal shunt placement); purple diamonds denote genetic investigations that returned negative results (karyotyping with array comparative genomic hybridization [array-CGH], and trio-based next-generation sequencing performed under a national rare disease research initiative); and green stars denote the molecular studies that established the diagnosis (whole-genome sequencing and parental Sanger confirmation). Background shading marks the three phases of the diagnostic journey. The orange-shaded section represents the perinatal and childhood period, during which the clinical course was dominated by hydrocephalus, ventriculoperitoneal shunt placement, and a working diagnosis of cerebral palsy. The blue-shaded section represents the prolonged diagnostic odyssey, during which the patient remained without a syndromic diagnosis despite undergoing three rounds of genetic testing. The green-shaded section represents the final diagnostic phase, during which whole-genome sequencing followed by Sanger confirmation identified a de novo likely pathogenic ABCC9 variant consistent with Cantú syndrome.

The diagnosis rests on the features that are established manifestations of Cantú syndrome: generalized hypertrichosis, coarse facial appearance, and cardiovascular involvement (mild aortic regurgitation and abnormal pulmonary vasculature), together with skeletal findings of bilateral hallux valgus and mild thoracic scoliosis, and the lower-extremity hyperpigmentation with intermittent peripheral edema that is consistent with the lymphatic and vascular involvement described in the syndrome. Combined with a de novo heterozygous *ABCC9* p.(Ile1392Val) variant, these support a diagnosis of Cantú syndrome (OMIM #239850; *ABCC9*-related hypertrichotic osteochondrodysplasia). Other findings in this patient—polycystic-appearing ovaries, oligomenorrhea, the renal echogenicity changes with small subcapsular cysts, and iron-deficiency anemia—are not established features of Cantú syndrome, and we do not claim them as such. They are common in the general population, and we cannot distinguish a coincidental co-occurrence from a genuine, previously unrecognized association on the basis of a single patient. We report them for completeness and because they required management, but they should not be read as expanding the phenotypic spectrum. Figure 7 summarizes the diagnostic trajectory from preterm birth in 1996 to molecular confirmation in 2026.

The patient and her family received post-test genetic counseling. The recurrence risk for future siblings was considered very low (<1%), accounting for the possibility of unrecognized parental germline mosaicism. The patient was also counseled regarding the 50% risk of transmission to future offspring, consistent with the autosomal dominant inheritance pattern of Cantú syndrome.

Multidisciplinary follow-up was arranged in accordance with current GeneReviews recommendations for Cantú syndrome and included cardiology for serial echocardiographic surveillance of valvular function and ventricular geometry; pulmonology and vascular imaging surveillance because of the previous episode of hemoptysis and known vascular involvement; ophthalmology; endocrinology/gynecology for management of polycystic ovaries and menstrual irregularities; orthopedics; and dermatology.

## 3. Discussion

The present case illustrates a form of diagnostic delay that is likely more common in clinical genetics than reflected in the literature: a patient with a major perinatal neurological event whose early clinical course becomes centered on management of that event, while manifestations of an underlying genetic syndrome gradually accumulate over subsequent decades. In this patient, hydrocephalus and the shunt-dependent neurological course appeared to provide a sufficient explanation for her developmental trajectory. Hypertrichosis, coarse facial features, hallux valgus, mild aortic regurgitation, polycystic ovaries, and the episode of hemoptysis in 2012 each had plausible isolated explanations at the time. Individually, none of these findings was sufficiently distinctive to trigger reconsideration of the underlying diagnosis. Only after the 2025 whole-genome sequencing result were these findings integrated into a coherent syndromic framework consistent with Cantú syndrome.

The differential diagnosis deserves comment, since the disorders raised in the Introduction share hypertrichosis and coarse facial features. Zimmermann–Laband syndrome is characterized by gingival fibromatosis and hypoplasia or aplasia of the nails and terminal phalanges, none of which was present here; our patient’s nails and distal phalanges were normal, and she had no gingival overgrowth. Coffin–Siris syndrome typically features hypoplasia of the fifth digit and nail, more severe intellectual disability, and a characteristic facial gestalt with sparse scalp hair—the opposite of the abundant scalp and body hair seen in this patient. The mucopolysaccharidoses were considered given the coarse facial appearance, but there was no organomegaly, corneal clouding, dysostosis multiplex, or joint stiffness of the type seen in those disorders, and the clinical course over three decades was not progressive in the expected way. Acromegaloid facial appearance syndrome lacks the cardiovascular and skeletal involvement documented here. What ultimately distinguishes Cantú syndrome in this patient is the combination of generalized hypertrichosis with cardiovascular involvement and the hemodynamic pattern attributable to reduced systemic vascular resistance, a combination not characteristic of the alternatives—together, of course, with the molecular finding.

An important question is why the trio-based next-generation sequencing study performed in 2019 failed to identify the ABCC9 variant, whereas whole-genome sequencing in 2025 detected it readily. Several factors likely contributed. First, Cantú syndrome was not clinically suspected at the time, so *ABCC9* was not specifically considered. The variant may therefore have been present in the raw sequencing data but excluded during filtering before clinical review. Because this testing was conducted within a national rare-disease research program rather than as a clinical diagnostic service, its gene content, filtering thresholds, and bioinformatic pipeline were retained internally and were not made available to the referring team, so the exact analytic workflow cannot be reconstructed here. Independently of the methodology, the variant would not have met the threshold for reporting at that time: it had no entry in ClinVar, was not supported by the gene–disease and in silico evidence then available, and—although parental samples were included in the 2019 trio—would have been regarded as a variant of uncertain significance rather than triggering the targeted de novo confirmation that such a program reserves for clearly pathogenic findings. Second, the period between 2019 and 2025 saw substantial advances in variant prioritization pipelines, expansion of population reference databases (including progression from gnomAD v2 to v4), and the introduction of improved computational missense prediction tools such as AlphaMissense, which were not routinely available in clinical practice in 2019.

We are cautious about what this case can be taken to show about sequencing modality. Because the platform, gene content, and filtering strategy used in 2019 were never disclosed to us, we cannot attribute the 2025 diagnosis to whole-genome sequencing as such. The variant is a single coding missense change that a well-analyzed exome would be expected to capture; what changed between 2019 and 2025 was not only the technology but the fact that the analysis was clinical rather than research-based, that the phenotype had by then accumulated, and that interpretation tools and population databases had advanced. The defensible conclusion is that renewed genomic investigation with contemporary interpretation was valuable—not that genome sequencing is inherently superior to exome sequencing in this setting. Reported diagnostic gains from systematic reanalysis of existing exome and genome data (approximately 10% in previously undiagnosed Mendelian disease [14], with a further 5–6% from automated reanalysis [15], and an incremental yield of roughly 7% on moving from exome to genome sequencing [16]) are consistent with this reading, and with the practical value of periodic reanalysis in patients whose clinical picture is dominated by an alternative, non-genetic working diagnosis.

The location of the variant is also of interest. *ABCC9* encodes the regulatory SUR2 subunit of K_ATP_ channels, which assemble with Kir6.1 or Kir6.2 pore-forming subunits and contain two cytoplasmic nucleotide-binding domains that dimerize on activation to form composite ATP-binding sites essential for gating [9,17]. Most reported Cantú syndrome–associated *ABCC9* variants cluster in the transmembrane domains or NBD1 [8,10]; p.(Ile1392Val) lies instead in NBD2. Structural modeling places Ile1392 on the β-strand that pairs with the Walker B strand, with its side chain buried in the hydrophobic core directly beneath the Walker B motif. Because Walker B contributes to the composite nucleotide-binding site formed on NBD dimerization, removing a methyl group from a tightly packed core position beneath it could in principle perturb the local packing on which that motif depends. This is a hypothesis, not a demonstrated mechanism: isoleucine-to-valine is a conservative change, the residue makes no direct contact with the catalytic carboxylates, and no functional data exist.

Variants in this region are thought to enhance Mg-nucleotide–dependent activation at the NBDs rather than to reduce ATP-mediated inhibition, the latter being conferred by the Kir6.1 pore-forming subunit. Functional characterization of p.(Gly814Trp), which lies in the corresponding signature motif of NBD1, showed that increased nucleotide binding and activation at the composite site can produce clinically significant gain-of-function effects [10]. Whether the same holds for position 1392 is unknown. No electrophysiological data are available for p.(Ile1392Val), and the substitution of isoleucine by valine is chemically conservative, which tempers any inference drawn from the residue’s location. The domain context makes a gain-of-function mechanism plausible; it does not establish one. Heterologous expression and patch-clamp studies will be required to determine whether this variant alters channel gating, and we regard the mechanism as inferential until such data exist.

Cantú syndrome has been reported predominantly in individuals of European or Latin American ancestry, a distribution that almost certainly reflects ascertainment and sampling bias rather than a true difference in prevalence. The International Cantú Syndrome Registry, currently the most comprehensive phenotypic dataset for the condition, includes 74 patients but no East Asian individuals [18]. Published Asian cases remain few: three Korean children with de novo ABCC9 variants [19], a Japanese family with autosomal dominant *ABCC9*-associated disease in which one affected adult developed an aortic aneurysm [20], and three Vietnamese children, also with de novo variants [21]. To our knowledge—based on a search of the published literature and of ClinVar, rather than on a formal systematic review—the present patient is the first molecularly confirmed Taiwanese case. Taken with the absence of East Asian patients from the registry, this suggests the condition is underrecognized in East Asian populations. Clinicians in Taiwan and neighboring regions should consider Cantú syndrome in patients with hypertrichosis, coarse facial features, and unexplained cardiovascular abnormalities, even when another diagnosis appears to account for the clinical course.

Therapeutic prospects have also evolved. Because the mechanism involves gain-of-function activation of K_ATP_ channels, the sulfonylurea glibenclamide has been an obvious candidate for repurposing. It reversed cardiovascular abnormalities almost completely in a knock-in mouse model [22], and a neonate carrying p.(Arg1116His) showed improved pulmonary hemodynamics on low-dose therapy, though escalation was limited by hypoglycemia [23]. Results in adults have been more sobering: Kleinendorst et al. reported reversal in a zebrafish model alongside the first open-label trial in adults, in which effects across eight participants were variable—modest improvement in hypertrichosis, but no significant change in cardiac phenotype or peripheral edema, and hypoglycemia again limiting dose escalation [24]. Whether more selective K_ATP_ inhibitors or variant-specific approaches will help remains unknown. For the present patient, now in her fourth decade with mild but established cardiovascular and reproductive manifestations, structured multidisciplinary surveillance rather than pharmacologic intervention is the immediate priority. The case nonetheless shows why a molecular diagnosis matters even in adulthood: it directs surveillance and may determine eligibility for future trials.

Several limitations should be acknowledged. The most important is that the pathogenic mechanism remains inferential. No functional characterization of p.(Ile1392Val) has been performed, so the gain-of-function behavior that defines Cantú syndrome has not been demonstrated for this variant; the classification rests on de novo occurrence, absence from population databases, partially concordant in silico evidence, and phenotypic fit (PS2, PM2_supporting, PP3_moderate, PP4), not on electrophysiological data. The substitution is chemically conservative, which makes direct functional evidence more, not less, necessary. We therefore cannot exclude the possibility that the variant will be reclassified—in either direction—as functional data, additional cases, or larger population datasets accumulate, and we regard the present classification as provisional in that sense. Heterologous expression with patch-clamp analysis of channel gating would be the appropriate next step. Second, this is a single case, so no inference about genotype–phenotype correlation is possible. Third, because of the patient’s substantial perinatal central nervous system injury, we cannot determine how much of her cognitive impairment and left hemiparesis is attributable to Cantú syndrome, although mild intellectual disability has been reported in a subset of patients [3,18]. Fourth, the 2012 hemoptysis and the associated pulmonary vascular abnormalities were reviewed retrospectively from historical records, and the original angiographic images were unavailable for reinterpretation. Finally, several findings in this patient are of uncertain relationship to the syndrome, as discussed above, and the details of the 2019 sequencing study were not available to us.

Despite these limitations, this case provides two important practical messages. First, Cantú syndrome may remain unrecognized into adulthood even in individuals with relatively classic phenotypic findings when systemic manifestations are mild or overshadowed by other major diagnoses. Second, periodic resequencing and reanalysis—particularly the transition from targeted research panels to clinical whole-genome sequencing using contemporary variant interpretation frameworks—can provide substantial diagnostic value in patients with long-standing undiagnosed disorders despite multiple previously negative genetic evaluations.

## 4. Conclusions

This case adds a single Taiwanese patient to a sparse East Asian Cantú syndrome literature and, more practically, demonstrates that periodic resequencing on contemporary platforms remains a high-yield step in the workup of long-standing undiagnosed disease, even after multiple earlier rounds of negative testing. The novel p.(Ile1392Val) variant within the SUR2 NBD2, immediately adjacent to the ABC signature motif, expands the mutational spectrum of *ABCC9* into a region where pathogenic variants remain comparatively sparse and where functional characterization may help clarify the underlying gating defect. For the patient herself, molecular confirmation has shifted the management plan from undirected neurological follow-up to structured multidisciplinary surveillance appropriate for her actual underlying condition; it has also opened the door to potential future participation in trials of targeted K_ATP_ channel modulation, should more selective or variant-tailored agents become available.

## Data Availability

The datasets generated and analyzed during the current study are not publicly available due to patient privacy considerations and restrictions imposed by the Institutional Review Board of MacKay Memorial Hospital. De-identified data supporting the findings of this study may be made available by the corresponding author on reasonable request, subject to approval by the Institutional Review Board.
